# Massage, and Allied Methods of Treatment: An Abstract of Lectures to Trained Nurses and Masseuses

**Published:** 1889-03

**Authors:** 


*Massage, and Allied Methods of Treatment: an Abstract of Lectures to Trained Nurses and Masseuses.* By HERBERT TIBBITS, M.D. Pp. 142. London: J. & A. CHURCHILL. 1888.

Within the scope permitted, and before the audience specially addressed—trained nurses,—these lectures must be regarded as successfully fulfilling their aim. The author's "design has been to cordially recognise all good work all over the world upon the subjects upon which he lectured; and not to arrogate to himself the position of *the* sole high priest of massage." We are glad to know this; and knowing it, our commendation of Dr. Tibbits' work is none the less hearty. Although these lectures were specially prepared for trained nurses and masseuses, they may be perused with pleasure and profit by trained and cultured medical men.

